# Myopic progression in school-aged children with moderate intermittent exotropia

**DOI:** 10.3389/fped.2023.1192387

**Published:** 2023-08-16

**Authors:** Tao Shen, Mintong Liang, Linxing Chen

**Affiliations:** State Key Laboratory of Ophthalmology, Guangdong Provincial Key Laboratory of Ophthalmology and Visual Science, Guangdong Provincial Clinical Research Center for Ocular Diseases, Zhongshan Ophthalmic Center, Sun Yat-Sen University, Guangzhou, China

**Keywords:** intermittent exotropia, myopic progression, strabismus surgery, school-aged children, spherical equivalent

## Abstract

**Objective:**

It is still controversial whether intermittent exotropia (IXT) affects myopic progression during the critical period of visual development. This study retrospectively analyzed the long-term myopic changes and the impact of IXT surgery on myopic progression in school-aged children with moderate IXT.

**Methods:**

The medical records of 65 children from 5 to 13 years old with or without IXT between 2015 and 2021 were retrospectively reviewed. Patients whose spherical equivalent refraction (SER) were less than −3.00 diopter (D) were included and divided into three groups: IXT surgery group (Group A), which comprised 22 IXT patients who received IXT surgery, IXT observation group (Group B), which comprises 19 IXT patients who only received long-term observational follow-up; and normal control group (Group C), which comprised 24 normal controls without IXT. The main outcome measurement was the rate of myopic progression, which was defined as the mean myopic shift in SER per year.

**Results:**

The 3- and 5-years long-term follow-up rates of myopic progression were −0.47 ± 0.28 D per year and −0.48 ± 0.23 D per year respectively in Group B, and those were significantly slower than that in Group C (−0.73 ± 0.32 D per year and −0.76 ± 0.19 D per year respectively). However, there was no significant difference in the rate of myopic progression between Group A and B or between Group A and C.

**Conclusion:**

Moderate IXT may have lower rate of myopic progression in school-aged children. Whether IXT surgery influence the rate of myopic progression still needs further study.

## Introduction

In school-aged children, myopia is the most common ocular disorder with increasing prevalence throughout the world and especially in Asia (1, 2), while intermittent exotropia (IXT) is the most common type of ocular misalignment in Asian countries (3, 4). While we know that esotropia is associated with hyperopia due to accommodation (5), the myopic progression in patients with IXT has not been rigorously studied. The role of accommodation in myopic progression has been continuously debated (6), and the increased accommodative demand to control the exodeviation in patients with IXT has been reported as a potential risk factor of myopic progression (7, 8). However, previous studies showed no correlation between myopic progression and IXT, and surgical correction of IXT did not alter the myopic progression (9, 10). But recent studies identified that the IXT surgery result in a faster myopic progression in the operated eye (11), and the non-dominant eye of IXT patient had a faster myopic progression (12). Additionally, it is still controversial to choose surgical correction of strabismus or not for moderate IXT patients (13), and the present study may provide a side evidence for this controversy in the perspective of myopia control.

Given that the association between myopic progression and IXT has not been established as well, the present study was conducted to investigate the relationship between moderate IXT and myopic progression and the impact of IXT surgery on myopic progression in school-aged children.

## Subjects and methods

This retrospective study was approved by the Institutional Review Board of Zhongshan Ophthalmic Center, Sun Yat-sen University (2015MEKY055) and followed the tenets of the Declaration of Helsinki.

The medical records of consecutive school-aged children from 5 to 13 years old at the time of first visit in our department of strabismus with moderate IXT between January 2015 and January 2021 were retrospectively reviewed. The moderate IXT was defined as a minimum intermittent exodeviation of 15 prism diopters (PD) at distance with normal simultaneous perception documented using synoptophore (13). All patients had spherical equivalent refraction (SER) between +2.00 and −3.0 diopter (D) in both eyes at the time of first visit. The following exclusion criteria were applied: anisometropia is greater than 3.00 D between two eyes; astigmatism is greater than 3.00 D in any eye; amblyopia; any other combined strabismus; previous history of strabismus surgery; previous history or plan of myopia control treatment or binocular vision training; neurologic or systemic disorder. The patients with moderate IXT were divided into two groups: Group A consisted of the patients who underwent strabismus surgery, and Group B consisted of the patients who were followed-up for observation. The control group (Group C) was matched for age and refraction, and consisted of children with orthotropia determined by the cover test.

All subjects included in this study underwent a complete ophthalmic examination and cycloplegic refraction annually for 5 years. Cycloplegic refraction was measured by subjective refraction following the topical administration of 1% tropicamide, and full corrections of refractive errors were prescribed. Ocular dominance was determined by the hole-in-the card test as previously described (14). Unilateral recession-resection surgery was performed on non-dominant eye in all subjects of Group A right after the initial presentation.

The main outcome measurement for myopic progression was the myopic shift rate, which was defined as the mean SER change per year (11). The interocular differences of myopic progression were analyzed for the comparison between right eye and left eye, between dominant eye and non-dominant eye, and between operated eye and non-operated eye, respectively (Supplementary Table S1). As there was no significant difference detected between two eyes, the average SER of two eyes were obtained for each subject and used for further comparative analysis of myopic shift rates among three groups.

### Statistical analysis

Statistical analysis was performed using the Statistical Package for Social Sciences program (version 22.0, SPSS Inc, Chicago, Illinois, USA). Means and standard deviations (SD) along with ranges were used to describe the clinical features in Tables 1, 2. Levene's test, Kruskal–Wallis test, and Mann–Whitney *U*-test were performed to evaluate differences between groups.

**Table 1:** Baseline demographic characteristics and spherical equivalent refraction changes of studied subjects in different groups.

			IXT surgery (Group A, *n* = 22)	IXT observation (Group B, *n* = 19)	Control (Group C, *n* = 24)	*P* Value		
						A vs. B	A vs. C	B vs. C
Gender (male/female)			6/16	6/13	11/13	0.76	0.06	0.14
Age (year)			8.09 ± 2.47	8.63 ± 3.02	7.67 ± 0.48	0.52	0.49	0.18
Spherical equivalent refraction (D)	Baseline		−0.18 ± 1.62	−0.28 ± 1.59	−0.02 ± 0.82	0.64	0.70	0.31
	Follow-up (year)	0.5	−0.48 ± 1.79	−0.45 ± 1.70	−0.33 ± 0.87	0.94	0.39	0.41
		1	−1.18 ± 1.71	−0.83 ± 1.71	−0.69 ± 1.03	0.86	0.56	0.71
		2	−1.18 ± 2.19	−1.22 ± 1.63	−1.56 ± 1.23	0.60	0.09	0.20
		3	−2.17 ± 2.45	−1.81 ± 1.62	−2.30 ± 1.36	0.54	0.69	0.16
		4	−2.23 ± 2.47	−2.58 ± 1.50	−2.98 ± 1.39	0.19	0.07	0.27
		5	−3.68 ± 2.88	−2.75 ± 2.03	−3.50 ± 1.16	0.28	0.91	0.12

IXT, intermittent exotropia; D, diopter.

**Table 2:** Myopic shift rates of studied subjects in different groups.

			IXT surgery (Group A, *n* = 22)	IXT observation (Group B, *n* = 19)	Control (Group C, *n* = 24)	*P* Value		
						A vs. B	A vs. C	B vs. C
Myopic shift rate (D/year)	Follow-up (year)	1	−0.94 ± 0.55	−0.50 ± 0.36	−0.83 ± 0.54	0.01*	0.16	0.07
		2	−0.58 ± 0.48	−0.49 ± 0.29	−0.74 ± 0.44	0.32	0.31	0.03*
		3	−0.74 ± 0.48	−0.47 ± 0.28	−0.73 ± 0.32	0.06	0.92	0.00**
		4	−0.64 ± 0.39	−0.65 ± 0.38	−0.73 ± 0.25	0.99	0.38	0.32
		5	−0.61 ± 0.33	−0.48 ± 0.23	−0.76 ± 0.19	0.16	0.97	0.00**

IXT, intermittent exotropia; D, diopter.

*p < 0.05; **p < 0.01.

## Results

**Figure 1:**
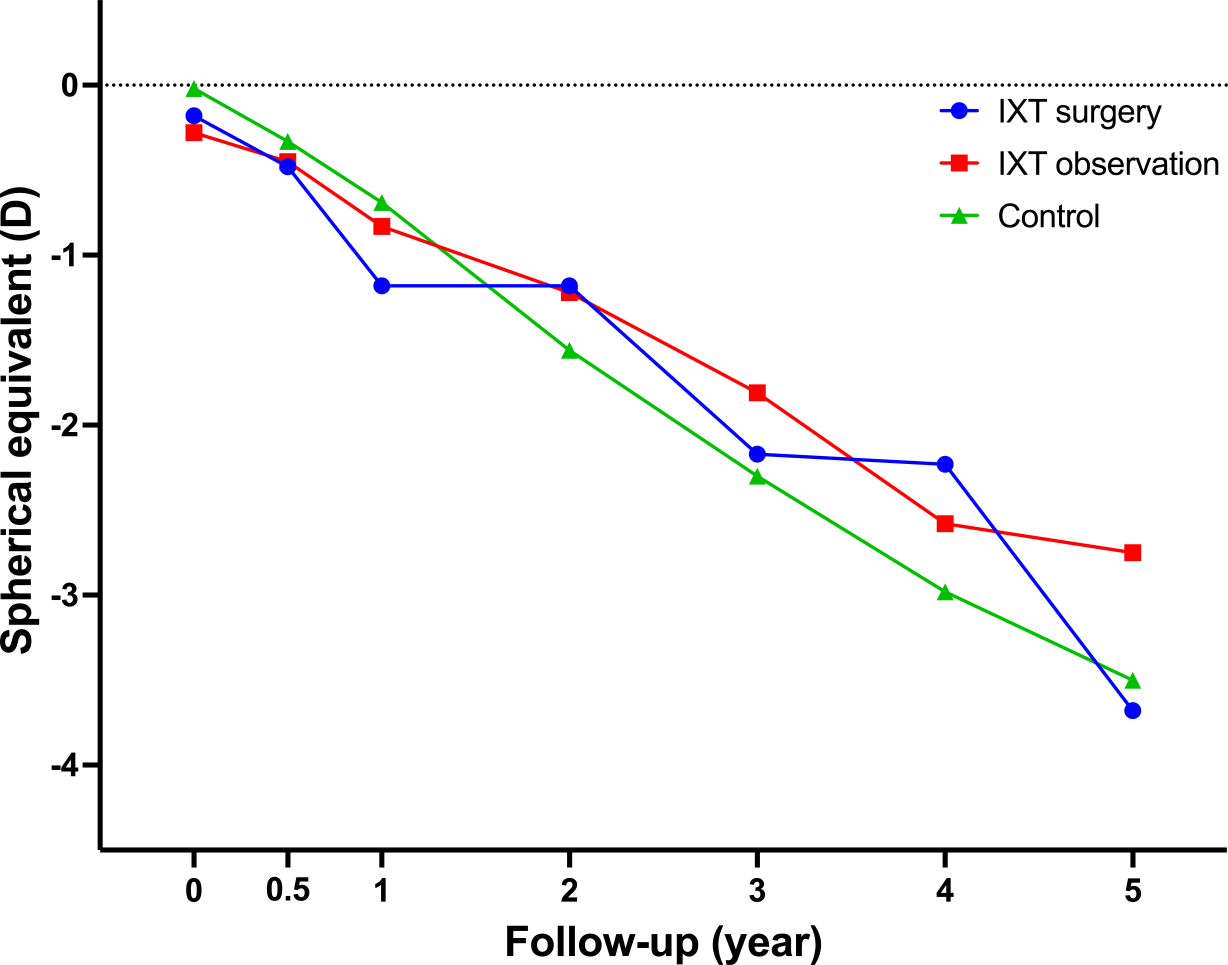
The changes of myopic status in the three studied groups. All three groups showed similar myopic shift without statistically significant differences during the follow-up period. IXT, intermittent exotropia; D, diopter.

A total of 65 school-aged children from 5 to 13 years old with or without IXT were included in this retrospective study. The IXT surgery group (Group A) comprised 22 patients with moderate IXT who received strabismus surgery, the IXT observation group (Group B) comprised 19 patients with moderate IXT who only received long-term observational follow-up, and the control group (Group C) comprised 24 normal subjects without IXT. The baseline demographic characteristics showed no significant intergroup difference in gender and age (Table 1). The initial mean SER were similar in three studied groups, which were −0.18 ± 1.62 D, −0.28 ± 1.59 D, and −0.02 ± 0.82 D, respectively. No intergroup difference of SER was detected at each follow-up evaluation either, although myopic progressions were detected in all studied groups. The changes of myopic status in the three studied groups were shown in Figure 1.

**Figure 2:**
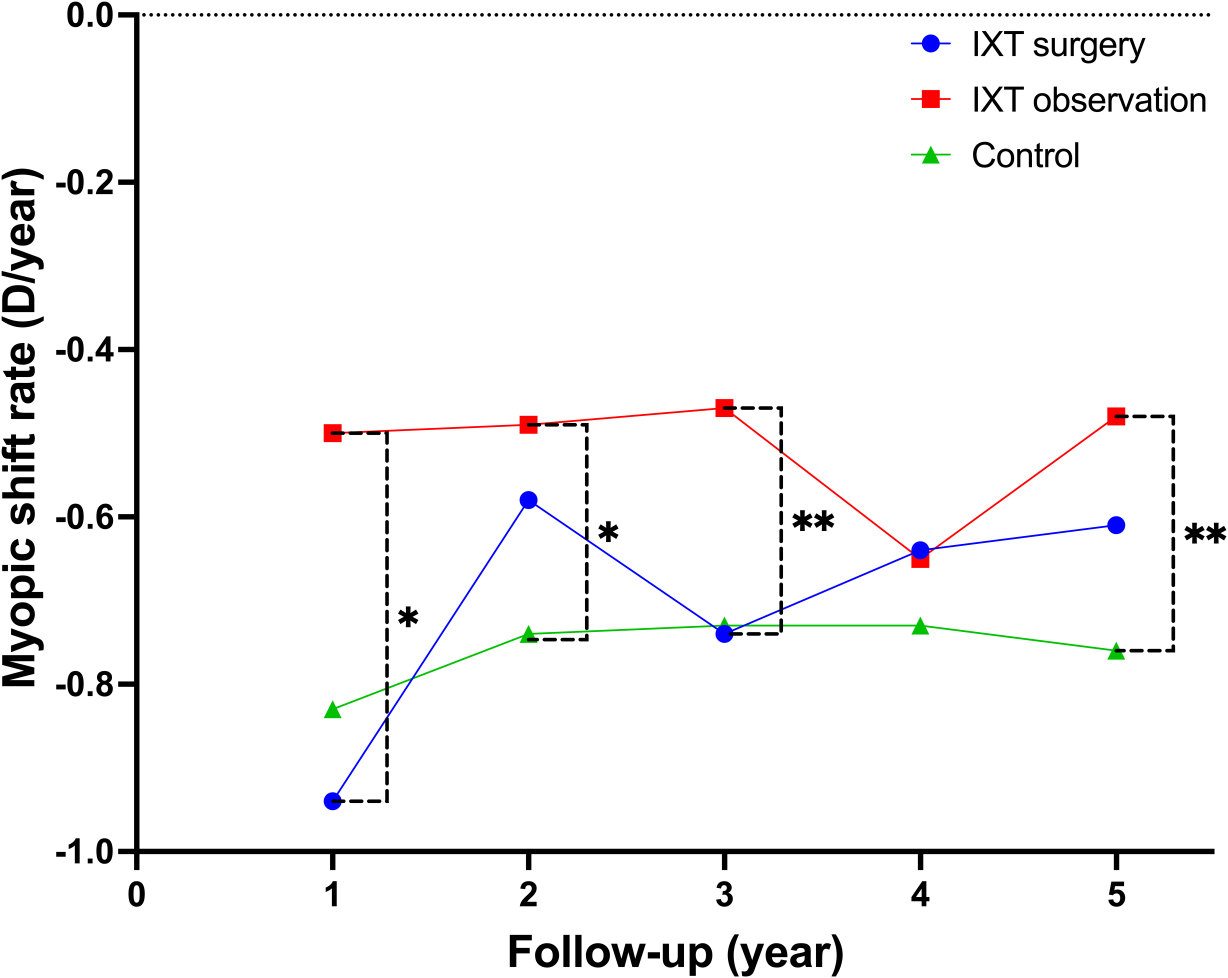
The changes of myopic shift rates in the three studied groups. Statistically significant differences in the mean myopic shift rates were detected between IXT observation group and control group at 2-, 3-, and 5-year follow-up. On the other hand, a statistically significant difference in the mean myopic shift rate was detected between IXT surgery group and IXT observation group only at 1-year follow-up. IXT, intermittent exotropia; D, diopter.

The myopic shift rates of studied groups at each follow-up are shown in Table 2. Between two groups (Group B and C) without surgical intervention, the mean myopic shift rates in patients with moderate IXT (Group B) were significantly lower than in normal controls (Group C) at 2-year (*p* = 0.03), 3-year (*p* < 0.001), and 5-year (*p* < 0.001) follow-up evaluation. In the patients with moderate IXT (Group A and B), the mean myopic shift rate in patients who underwent strabismus surgery (Group A) was significantly higher than the observation group (Group B) at early postoperative follow-up (*p* = 0.01 at 1-year follow-up), however, no difference was noted in the mean myopic shift rates between these two groups subsequently. The changes of myopic shift rates in the three studied groups are shown in Figure 2.

## Discussion

The present retrospective study on 65 school-aged children showed a significant overall myopic progression in all studied groups. Although we failed to find a statistically significant intergroup difference of SER during follow-up period, we detected intergroup differences in myopic shift rates between Group A and Group B (*p* = 0.01 at 1-year) at early postoperative follow-up and between Group B and Group C (*p* = 0.03 at 2-year, *p* < 0.001 at 3-year, and *p* < 0.001 at 5-year) in long-term follow-up. These findings are not consistent with previous studies (9, 10), which reported that IXT itself or surgical correction of IXT did not alter the myopic progression.

In the present study we set the inclusion criteria of age as 5–13 years old, and the mean ages of three studied groups were 8.09 ± 2.47, 8.63 ± 3.02, and 7.67 ± 0.48 respectively. Simple myopia usually occurs in early school-age of 5–13 years old, and the myopic progression has been reported to cease at 15–16 years of age (15–17). So, the school-age is the most critical period for the progression of myopia. Besides, the refractive status of the enrolled subjects in all three studied groups were mild myopia at initial presentation. In view of children with myopia showed faster myopic progression (18), we investigated myopic shift rates in school-aged children with mild myopia, in order to identify the impact of IXT and surgical correction of IXT on myopic progression in these specific population. In addition, we chose the children only with moderate IXT who manifest obvious exodeviation frequently but with only partial impairment of binocular single vision, and it was different from previous studies containing a various severity of IXT (9, 10).

No significant difference detected between dominant eye and non-dominant eye among three studied groups in our study, however, it has been reported that the non-dominant eye had a faster myopic progression which associated with severe IXT caused by greater gap between the required accommodative demand and response (12). The results of our study can be explained by the specific type of moderate IXT patients which included in the present study, and the impact of moderate IXT on the myopic progression of the non-dominant eye might be not as strong as severe IXT. In contrast, the moderate IXT children in Group B showed slow myopic progression than the normal control in Group C. The moderate IXT children had relatively better control of exodeviation, which indicated that sufficient accommodative response compared with accommodative demand in these moderate IXT children to control normal ocular alignment. The mechanism involved in the control of IXT is still of limited understanding (19), and coupled accommodation and vergence responses are needed for compensating the additional convergence demand when overcoming the exodeviation to achieve normal alignment in IXT patients (20). The control of IXT is typically driven by neither accommodative effort alone nor convergence effort alone. In moderate IXT patients with good control, over-accommodation secondary to fusional convergence may easily overcome the gap between the required accommodative demand and actual accommodative response. So, for the moderate IXT children in the present study, achieving normal alignment did not conflict with having clear image on the retina, and possibly resulted in reduction of the myopic shift rates. The impact of the moderate IXT on myopic progression has been reconfirmed by removing this probable protective factor, the myopic shift rates remained unchanged after surgical correction of IXT (Group A vs. Group C).

The previous studies reported a significant myopic progression in the operated eye following surgical correction of IXT in early school-aged children (11, 21, 22), although other reports showed no significant change in myopic progression after IXT surgery (10, 23). A faster myopic shift in the operated eye might be caused by either surgical procedure or accommodative lag in the non-dominant eye. In present study, we detected a transient myopic shift induced by IXT surgery (Group A vs. Group B at 1-year), however, no significant difference was detected between operated eye and non-operated eye in Group A. So, it is still unclear whether the IXT surgical procedure or surgical removal of IXT is responsible for the transient myopic change.

The limitations of the present study are as follows. First, a relatively small sample size of the retrospective study induced small fluctuation in the changing curve of myopic shift rates (Figure 2). The sample size is approximately calculated as 110 if take the present study as a preliminary experiment, when the calculation assumes 5% type I error with 80% power. Second, we chose moderate IXT children to investigate the impact on myopic progression, but we did not analyze the effect in terms of the amount of deviation angle, the degree of control, the types of strabismus surgery, and the outcomes of surgical correction. Third, this trend has been observed in one regional province and may need larger studies before it can be applied globally.

The present study, which require confirmation in future, showed different results compared with previous studies in IXT patients (9, 10, 12), moderate IXT may have lower rate of myopic progression in school-aged children. Myopic progression is influenced by a number of factors (24). Therefore, the impact of IXT with different severity on myopic progression should be further analyzed considering various influencing factors, which may provide helpful evidences in determining the optimal choice for IXT treatments.

## Data Availability

The raw data supporting the conclusions of this article will be made available by the authors, without undue reservation.
