# Supplementary material for: Myopic progression in school-aged children with moderate intermittent exotropia
Source: Front Pediatr. 2023 Aug 16;11:1192387. doi: 10.3389/fped.2023.1192387 (PMC10466788; doi:10.3389/fped.2023.1192387)
Supplement: Supplementary file 1 [file Table1.docx]

**Supplementary Table 1. The Interocular differences of myopic shift rates in studied subjects.**

|  | | **IXT surgery**  **(Group A, n=22)** | **IXT observation**  **(Group B, n=19)** | **Control**  **(Group C, n=24)** |
| --- | --- | --- | --- | --- |
| Myopic shift rate (D/year) | Right eye | -0.53±0.33 | -0.42±0.30 | -0.76±0.22 |
|  | Left eye | -0.61±0.32 | -0.54±0.23 | -0.78±0.26 |
|  | P value | 0.11 | 0.08 | 0.24 |
|  | Dominant eye | -0.78±0.32 | -0.60±0.32 | -0.78±0.30 |
|  | Non-dominant eye | -0.80±0.45 | -0.54±0.19 | -0.74±0.27 |
|  | P value | 0.95 | 0.95 | 0.23 |
|  | Operated eye | -0.56±0.38 | NA | NA |
|  | Non-operated eye | -0.66±0.35 | NA | NA |
|  | P value | 0.14 | NA | NA |

IXT = intermittent exotropia; D = diopter.
